# Re-examining the association between residential exposure to magnetic fields from power lines and childhood asthma in the Danish National Birth Cohort

**DOI:** 10.1371/journal.pone.0177651

**Published:** 2017-05-17

**Authors:** Madhuri Sudan, Onyebuchi A. Arah, Thomas Becker, Yael Levy, Torben Sigsgaard, Jørn Olsen, Ximena Vergara, Leeka Kheifets

**Affiliations:** 1 Department of Epidemiology, UCLA Fielding School of Public Health, Los Angeles, California, United States of America; 2 Department of Clinical Epidemiology, Aarhus University, Aarhus, Denmark; 3 College of Osteopathic Medicine of the Pacific, Western University of Health Sciences, Pomona, California, United States of America; 4 Department of Environmental Science, Aarhus University, Aarhus, Denmark; 5 Kipper Institute of Immunology and Allergy, Schneider Children’s Medical Center of Israel, Petach Tikvah, Israel; 6 Department of Public Health, Aarhus University, Aarhus, Denmark; 7 Environment Sector, Electric Power Research Institute (EPRI), Palo Alto, California, United States of America; Stony Brook University, Graduate Program in Public Health, UNITED STATES

## Abstract

**Background:**

A study reported an increased risk of asthma in children whose mothers were exposed to magnetic field (MF) levels above 0.2 μT during pregnancy. We re-examined this association using data from mothers and children in the Danish National Birth Cohort (DNBC).

**Methods:**

This study included 92,676 singleton-born children and their mothers from the DNBC. MF exposure from power lines was estimated for all residences where the mothers lived during pregnancy and for all children from birth until the end of follow up. Exposure was categorized into 0 μT, 0.1 μT, and ≥ 0.2 μT for analysis. Definitive and possible asthma cases were identified using data from three independent data sources: 1) mothers’ reports, 2) a national hospitalization register, 3) a national prescription drug register. We calculated hazard ratios (HR) and 95% confidence intervals (CI) for the association between the highest level of exposure during pregnancy and asthma in children, adjusting for several potential confounding factors. We also examined the sensitivity of the risk estimates to changes in exposure and outcome definitions.

**Results:**

No differences or trends in the risk of asthma development were detected between children with different levels of MF exposure regardless of the asthma case definition or outcome data source. For definitive cases, the HR (95% CI) for those with any exposure was 0.72 (0.27–1.92), and it was 0.41 (0.06–2.92) for those exposed to ≥ 0.2 μT. Adjustments for confounding and variations in the exposure definition did not appreciably alter the results.

**Conclusion:**

We did not find evidence that residential exposure to MF during pregnancy or early childhood increased the risk of childhood asthma. This interpretation is in line with the lack of an established biological mechanism directly linking MF exposure to asthma, but high exposure was very rare in this cohort.

## Introduction

Exposure to extremely low frequency magnetic fields is ubiquitous. This has raised concerns among the public about its possible health effects. Associations between residential magnetic field (MF) exposures and childhood leukemia have been reported rather consistently across numerous epidemiologic studies over the past several decades, but the biological mechanism for an effect has not be been identified, and given the lack of biological plausibility at residential exposure levels, much uncertainty remains about the nature of these associations [1,2]. A number theories, including the “population-mixing hypothesis” and the “delayed infection hypothesis”, suggest that some childhood leukemias may be caused by infection and immune-related risk factors [3,4]. Childhood asthma is another condition that may be due to an anomalous immune response, and a relationship between asthma and childhood leukemia has been posited [5], which is unlikely to be entirely due to uncontrolled confounding from infections [6]. Thus, investigating the potential relationship between MF and asthma may help to shed light on the MF-leukemia association.

Asthma is the most common non-communicable disease among children and often poses a significant health burden [7]. In Denmark, several investigators have estimated an asthma prevalence of around 10% among children [8,9]. Like other chronic inflammatory diseases, asthma and allergic diseases result from interactions between genetic background, the immune system, and environmental factors. The exposure to various environmental factors depends on child lifestyles, which have changed greatly during the last few decades, and may affect the immune system through changes in the microbiome, and epigenetic factors [10,11].

More than 15 years ago, Beale et al. conducted a cross-sectional study to examine the relationship between the MF exposure of adults in their homes and prevalence of immune-related and other chronic illnesses [12]. Five hundred and sixty adults living near extra high voltage transmission lines completed questionnaires about their health and demographic characteristics. After adjustment for possible confounding, elevated odds ratios (OR) were observed at higher exposure levels for both asthma and combined chronic illnesses. Due to numerous severe weaknesses, such as potential self-reporting bias and lack of established temporality, Beale et al.’s study received little attention and no replication.

Employing a better design, a more recent study by Li et al. reported an increased risk of asthma in children whose mothers were exposed to higher levels of measured MF during pregnancy [13]. After adjustment for maternal age, race, education, smoking during pregnancy, and a maternal history of asthma, a linear dose-response relationship was observed between increasing maternal median daily MF exposure level in pregnancy and an increased risk of asthma in offspring (adjusted hazard ratio [aHR] per 0.1 μT increment, 1.15; 95% confidence interval [CI], 1.04–1.27). When exposures were assigned to 3 categories, with the low category (≤ 0.03 μT) used as the reference group, the authors observed an aHR of 1.74 (95% CI: 0.93–3.25) for medium exposure (≥ 0.03 to 0.2 μT) and 3.52 (95% CI: 1.68–7.35) for highest exposure (above 0.2 μT) for “definitive” asthma diagnosis. A similar, but weaker, association was seen among the suspected asthma cases, with aHRs of 1.24 and 1.41 for medium and high exposures, respectively. A much stronger association was observed in a subset of children whose mothers had a history of asthma.

The investigation by Li et al. was performed in a group of children whose mothers participated in a study aimed at examining associations between maternal exposure to extremely low frequency magnetic fields (ELF-MF) and miscarriage [14]. The mothers were asked to wear an EMDEX-II meter for 24 hours during the first or second trimester of pregnancy, and the cohort of children born from these pregnancies was followed up until 13 years of age. Of the 2,729 pregnant women eligible for the miscarriage study, 1,380 agreed to participate, of whom 1,063 were interviewed, and 829 delivered a live birth. After excluding participants that were lost to follow-up, did not have ELF-MF measurements, or whose asthma diagnosis was uncertain, there remained 626 participants for analysis in the asthma study.

While the study by Li et al. has the advantage of a cohort design, it also has major limitations. First, the definition of asthma diagnosis used in the study was potentially problematic. In order to be considered a case, the child must have received a clinical diagnosis of asthma on at least 2 occasions within one year. Thus, children who were heavier users of healthcare services for other reasons may have been more likely to be identified as cases. Second, the 24-hour MF measurements were only taken on a single day during the first or second trimester of pregnancy. These measurements could have been biased by time-specific conditions of pregnancy, such as morning sickness, that are likely to have altered the woman’s exposure [15]. Third, the analysis lacked control for some potentially important confounding factors, such as respiratory infections and exposure to tobacco smoke, which are thought to be related to asthma [16–18]. Finally, the rationale behind the choice of MF metrics (average versus highest encountered) and cut-points (above 0.2 μT) used in the asthma study versus what was used by the same investigators in their miscarriage study (above 1.6 μT) is not given and is not clear. Nevertheless, given the high prevalence of childhood asthma and the ubiquitous exposure to power frequency magnetic fields, a potential association may have significant potential public health impact and needs to be further investigated.

The present study aims to re-examine the association between exposure to magnetic fields during pregnancy and childhood asthma that was reported by Li, et al. using data from mothers and children in the Danish National Birth Cohort (DNBC). We aim not only to replicate the previous study by following their overall asthma and exposure categorizations, but also to overcome limitations of that study with clearer and more specific asthma case definitions, objective exposure assessment methods with exposure categories defined *a priori*, and adjustment for some additional potential confounding factors.

## Methods

The DNBC enrolled 91,661 pregnant women in Denmark between 1996 and 2002, with 9,380 enrolled again during subsequent pregnancies in the enrollment period. More than 95% of women in Denmark are registered with a General Practitioner (GP), and nearly all visit their GP during weeks 6–12 of pregnancy to confirm the pregnancy and receive a referral for follow-up care. Women were invited to participate in the DNBC by their GPs during this first prenatal visit. Half of all GPs participated in this process, Approximately 50% of all pregnant women in Denmark were invited, of which about 60% participated. This study focuses on the 92,675 children born into the cohort from singleton births. Details about the design of the DNBC were published previously [19].

The DNBC women and children have been followed since enrollment, and mothers have completed interviews and questionnaires at various prenatal and postnatal periods, including prenatal interviews at weeks 12 and 30 of gestation, early life interviews when the children were 6 months old and 18 months old, and again when the children were 7 years old. At each time point, mothers were asked to report detailed information on their health, lifestyle, and environmental exposures and those of their child. Information on social conditions, birth data, diagnoses made in connection with hospital and specialty care visits, and prescription drugs were obtained from Denmark’s various population registers. The study also recorded each mother’s residential address at the time her child was born, and obtained each child’s address in Denmark for every year after he or she was born.

### Exposure assessment

We obtained GPS coordinates for the front door of the home—or building in the case of an apartment—for all residences in which the DNBC mothers lived from the time of conception until the date of the child’s birth, and for all children from birth until the end of follow up. The GPS coordinates were obtained from a free database provided by the Agency for Data Supply and Efficiency (previously named The Danish Geodata Agency). Danish electrical utility companies provided the locations of all existing and historical 132–400 kV overhead transmission lines. The distance from the residence to the nearest power line that existed at the date of interest was calculated in ArcGIS 9.3.

The utility companies also provided estimated magnetic field levels within specific distances (buffers) of each power line as a categorical variable with exposure levels of 0 μT, 0.1 μT, 0.2 μT, or 0.4 μT. These estimates were based on line configurations, historical loading, phasing, and direction of current flow. For each home, and blind to disease status, we assigned magnetic field levels based on the value of the buffer into which the home fell. For homes falling into more than one buffer, the highest magnetic field level was assigned. Homes located outside of the buffer area for any power line were assigned an exposure level of 0 μT. Based on these assignments, we developed a categorical exposure variable indicating the highest exposure level during pregnancy, and another categorical exposure variable indicating the highest exposure level from the time of conception until the end of follow-up for the child. These variables were categorized into levels of 0 μT, 0.1 μT, and ≥ 0.2 μT for analysis, to allow for sufficiently large numbers in each category.

### Outcome assessment

Asthma cases were identified using data from three independent data sources. One source was the mothers’ reports of their child’s asthma diagnosis. In the DNBC age-7 questionnaire, mothers were asked to report whether or not a doctor had ever said her child had asthma. An answer of ‘yes’ to this question was considered an asthma diagnosis. A second source was the Danish National Patient Register (DNPR) [20]. The DNPR includes all medical diagnoses made during hospital admissions and visits to emergency rooms and specialty outpatient clinics in Denmark. All children are included in the DNPR from the time of birth. A diagnosis recorded in the DNPR with ICD-10 code J45, J45.0, J45.1, J45.8, J45.9, J46.0, or J46.9 was considered an asthma diagnosis. A third source was the Register of Medicinal Product Statistics (RMPS), which includes records of all prescription medications filled in Denmark [21]. In order to be considered an asthma diagnosis based on the RMPS data, a child must have filled prescriptions for anti-asthmatic medications with Anatomic Therapeutic Chemical (ATC) system codes R03A, R03B, R03C, or R03D at least two times. Liquid and tablet medications were excluded, as these are typically not prescribed for asthma.

For similarity with the Li et al. study, which excluded “suspected” asthma diagnosis (those who had either only one diagnosis, or two diagnoses that were more than one year apart, or those who used anti-asthmatic medications without a clinical diagnosis of asthma) for the main analysis, we included a “definitive asthma case” outcome in our analysis. A definitive asthma case in our study was a child who had an asthma diagnosis present in all of the three data sources. We also developed a “possible asthma case” outcome definition, which was defined as a child who had an asthma diagnosis in any of the three data sources.

### Statistical analysis

The association between MF exposure and development of asthma in children was examined using survival analysis methodology. Follow-up time for each child began at the start of the mother’s pregnancy (estimated by physician based on last menstruation) and ended (1) when the child became an asthma case, or (2) when he or she left Denmark or was no longer included in the DNPR and RMPS for other reasons (e.g., death). We tested the assumptions of the Cox proportional hazard model, and found that the ratio of the hazard functions for two observations with different values for the independent variable did not depend on time, and there was a log-linear relationship between the independent variables and the underlying hazard function.

We calculated HRs and 95% CIs for the association of interest using Cox proportional hazard models. Our main analysis related the highest level of exposure during pregnancy to the risk of asthma in children. We also examined the sensitivity of the risk estimates to changes in the exposure and outcome definitions. We did this by repeating our main analysis using a binary exposure variable (any exposure/no exposure), expanding the exposure time-period to include the time from conception to the end of follow-up, and examining asthma diagnosis from each outcome data source separately.

We considered a large number of potential confounding factors for adjustment in our regression models. Our final adjusted models included the child’s sex, exposure to environmental tobacco smoke in the home, birth order, breastfeeding, early life respiratory infections, other early-life infections, vaccinations, and exposure to animals outside the home, as well as maternal and paternal histories of asthma, combined parental social-occupational status, maternal age, and whether the mother lived on farm or with farm animals during pregnancy.

### Ethics

Mothers provided written informed consent prior to inclusion in the cohort. Mothers who requested to discontinue participation at any time or whose child was deceased were no longer contacted for further follow-up. This study was approved by the Danish Data Protection Agency, the regional science ethics committees in Denmark, and the Office for the Protection of Research Subjects at the University of California, Los Angeles.

## Results

Our study sample consisted of 92,675 children born to 91,661 mothers. Twelve percent of the 92,675 children had a respiratory infection recorded in the DNPR by age 3 years (Table 1). According to mothers’ reports, approximately 31% of children had at least one of six common childhood viral infections by the age of 18 months, and about 63% had received all of the 7 common childhood vaccinations by age 18 months. About 9% of mothers and 8% of fathers had a history of asthma according to mothers’ reports.

**Table 1:** Characteristics of study sample.

	N (%)
Child’s sex	
Male	47,498 (51.3)
Female	45,177 (48.7)
Child exposed to tobacco smoke in home before 18 months	27,344 (29.5)
Child’s birth order	
1	40,704 (43.9)
2	32,073 (34.6)
3+	13,952 (15.1)
Child was breastfed	52,264 (56.4)
Child had respiratory infection in DNPR before age 3	11,082 (12.0)
Child had other common childhood infection before age 18 months	
Any below	28,375 (30.6)
Chicken pox	12,885 (13.9)
Measles	292 (0.3)
Mumps	62 (0.07)
Rubella	382 (0.4)
Scarlet fever	1,084 (1.2)
Roseola infantum	18,285 (19.7)
Child received all 7 common vaccinations by age 18 months	58,448 (63.1)
Child in regular contact with animals outside of home before age 18 months	17,726 (19.1)
Mother has history of asthma	7,882 (8.5)
Father has history of asthma	7,107 (7.7)
Social occupational status	
High	57,737 (62.3)
Medium	52,236 (27.2)
Low	3,480 (3.8)
Maternal age at birth of child	
<26	11,239 (12.1)
26–30	37,694 (40.7)
31–35	32,237 (34.8)
>35	11,505 (12.4)
Mother smoked during pregnancy	23,774 (25.7)
Mother lived on farm or with farm animals while pregnant	5,947 (6.5)

**Fig 1:**
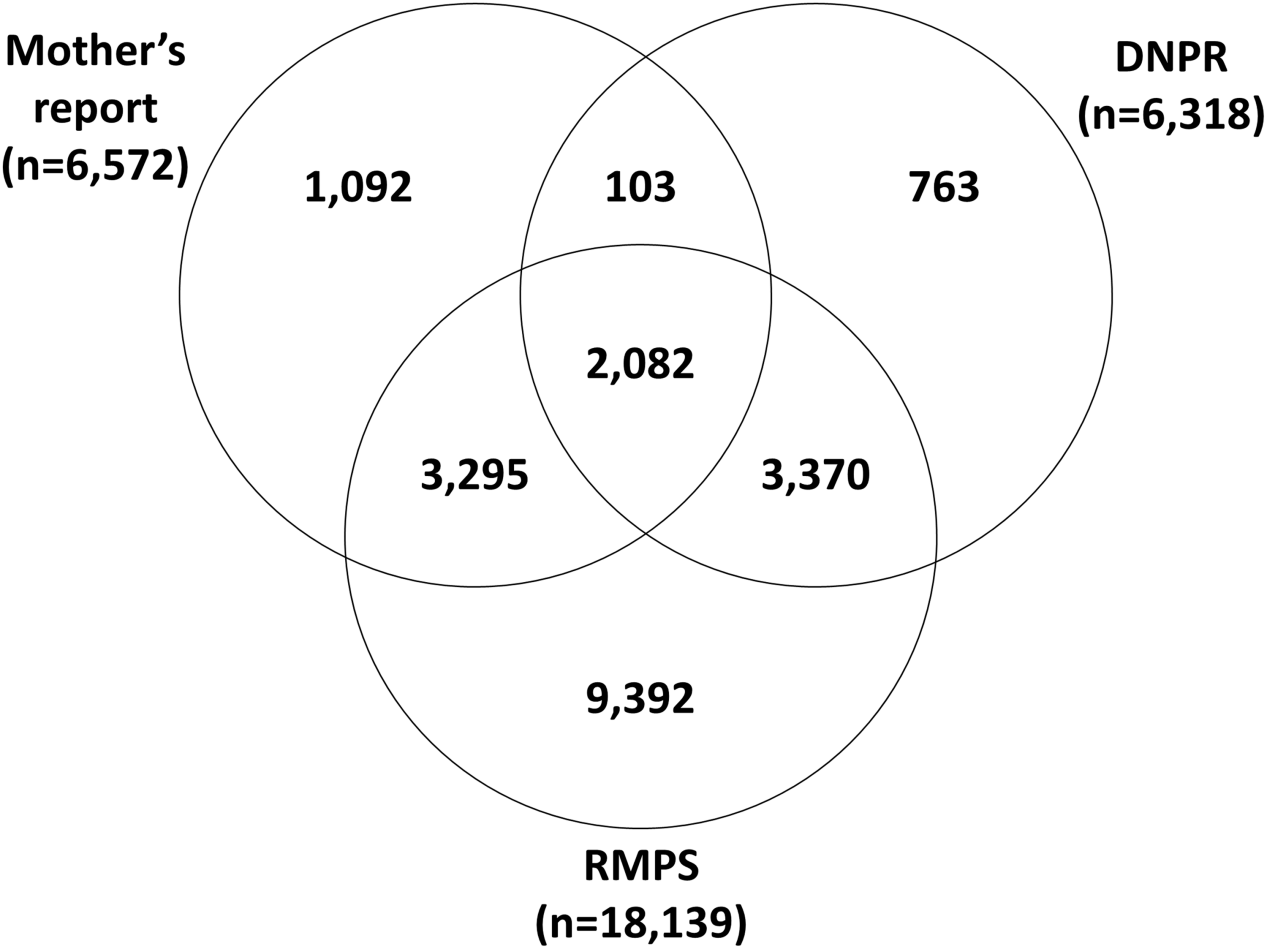
Distribution of asthma cases by outcome source.

We identified 2,082 (2%) definitive cases and 20,097 (22%) possible cases of asthma in this study (Table 2, Fig 1). When each outcome data source was examined separately, we found 6,572 (12%) children who were diagnosed with asthma according to mothers’ reports, 6,318 (7%) cases based on the DNPR, and 18,139 (20%) based on the RMPS. Average age of diagnosis was lowest (3.1 years) for those children who were definitive cases.

**Table 2:** Distribution of asthma cases and age at first diagnosis by data source and outcome definition.

	N (%)	Mean (SD) age at first diagnosis
Asthma cases by data source		
Mother’s report (DNBC Age 7 Questionnaire)	6,572 (12.0)	7.2* (0.14)
DNPR (Hospital Register)	6,318 (6.8)	3.8 (3.5)
RMPS (Prescription Register)	18,139 (19.6)	5.1 (4.4)
Possible case (Any outcome source)	20,097 (21.7)	4.5 (4.0)
Definite case (All three outcome sources)	2,082 (2.3)	3.1 (2.2)

* Approximate age of child at the time when mother reported child “ever diagnosed with asthma”

The vast majority of mothers and children in our dataset had no residential MF exposure from powerlines (Table 3). Among possible cases, only 24 (0.12%) had mothers who were exposed during pregnancy and only 38 (0.19%) were exposed at any time during follow-up. Exposure was similarly low for non-cases.

**Table 3:** Distribution of MF exposure by time period and possible case status*.

Highest exposure level	Exposure during pregnancy		Exposure from conception until diagnosis/end of follow-up	
	Case N (%)	Non-Case N (%)	Case N (%)	Non-Case N (%)
≥ 0.2 μT	7 (0.03)	38 (0.05)	13 (0.06)	84 (0.12)
0.1 μT	17 (0.09)	56 (0.08)	25 (0.12)	104 (0.14)
No exposure	19,990 (99.88)	71,673 (99.87)	20,059 (99.81)	72,390 (99.74)
Binary				
Any exposure	24 (0.12)	94 (0.13)	38 (0.19)	188 (0.26)
No exposure	20,073 (99.88)	72,484 (99.87)	20,059 (99.81)	72,390 (99.74)

* Identified as an asthma case by any of the three outcome sources

We did not detect differences or trends in the risk of asthma development between children with different levels of exposure based on the Cox proportional hazard models. However, the results had wide confidence intervals. This was true for the analyses using the possible and definitive case definitions of asthma (Table 4) as well as for the analyses using asthma diagnoses defined separately by each individual data source (Table 5). Except for definite cases with any exposure and definite cases with exposure level of 0.1 μT, adjustment for confounding and variations in the specification of the exposure variable did not appreciably alter the imprecise results (Table 4). Adjusted and unadjusted results were similar across individual data sources. However, when the outcome was defined by hospital register data only, the results moved positively away from null after adjustment at the 0.1 μT exposure level, and towards the null when the outcome was defined by the questionnaire or prescription drug data for the same exposure level (Table 5).

**Table 4:** Hazard ratios (HR) for asthma in relation to MF exposure at any time and asthma diagnosis by possible and definitive outcome.

Highest exposure from conception until end of follow-up	Possible case		Definite case	
	HR (95% CI)	Adjusted HR (95% CI)*	HR (95% CI)	Adjusted HR (95% CI)*
≥ 0.2 μT	0.59 (0.34–1.01)	0.69 (0.36–1.34)	0.41 (0.06–2.92)	0.59 (0.08–4.20)
0.1 μT	0.86 (0.58–1.28)	0.93 (0.55–1.58)	0.96 (0.31–2.98)	1.98 (0.64–6.15)
No exposure	1.00	1.00	1.00	1.00
Binary				
Any exposure	0.74 (0.54–1.02)	0.82 (0.55–1.24)	0.72 (0.27–1.92)	1.25 (0.47–3.33)
No exposure	1.00	1.00	1.00	1.00

*Adjusted for sex, mother’s and father’s history of asthma, social-occupational status, maternal age, smoking during pregnancy, child’s exposure to environmental tobacco smoke in the home, breastfeeding, early childhood respiratory infections, other common childhood infections, vaccination, mother lived on farm or with farm animals during pregnancy, child’s exposure to animals outside home, and birth order.

**Table 5:** Associations between MF exposure from conception until diagnosis/end of follow-up and asthma diagnosis by individual data source.

Highest exposure level	Age-7 Questionnaire		DNPR Hospital Register		RMPS Prescription Register	
	HR (95% CI)	Adjusted HR (95% CI)*	HR (95% CI)	Adjusted HR (95% CI)*	HR (95% CI)	Adjusted HR (95% CI)*
≥0.2 μT	1.22 (0.61–2.44)	1.03 (0.46–2.29)	0.54 (0.21–1.44)	0.63 (0.20–1.95)	1.11 (0.74–1.67)	1.16 (0.69–1.97)
0.1 μT	1.45 (0.83–2.56)	1.15 (0.55–2.42)	1.04 (0.56–1.93)	1.42 (0.64–3.17)	1.13 (0.79–1.61)	1.03 (0.61–1.75)
No exposure	1.00	1.00	1.00	1.00	1.00	1.00
Binary						
Any exposure	1.35 (0.87–2.09)	1.09 (0.63–1.88)	0.82 (0.49–1.39)	1.00 (0.52–1.93)	1.12 (0.86–1.46)	1.10 (0.76–1.59)
No exposure	1.00	1.00	1.00	1.00	1.00	1.00

*Adjusted for sex, mother’s and father’s history of asthma, social-occupational status, maternal age, smoking during pregnancy, child’s exposure to environmental tobacco smoke in the home, breastfeeding, early childhood respiratory infections, other common childhood infections, vaccination, mother lived on farm or with farm animals during pregnancy, child’s exposure to animals outside home, and birth order.

## Discussion

We aimed to replicate and improve upon the study by Li et al. and re-examine their reported association between exposure to magnetic fields during pregnancy and childhood asthma. We did not detect an association between residential MF exposure and childhood asthma regardless of asthma definitions or confounding adjustment at the levels of exposure reported by Li et al., but low exposure prevalence limits our ability to make firm conclusions.

Asthma is a heterogeneous disease, often lacking standard disease definitions. The strengths of our study include the evaluation of multiple asthma case definitions, which were based on several complete and high quality data sources. Each source may have limitations (e.g., hospital registers capture acute events which might be severe), but overall, discharge diagnoses of asthma appear well-correlated (91%) with medical diagnosis [22]. A previous study using the same approach to examine asthma in the same population found substantial non-overlap between data sources [23]. Nonetheless, our approach allowed for comparisons across data sources, specified *a priori*.

Additionally, we used objective exposure assessment methods that captured MF exposures due to overhead powerlines during both pregnancy and early childhood. We developed a detailed analysis plan with exposure categories defined *a priori*. Our analysis included adjustment for numerous confounding factors, including, importantly, family history, exposure to allergens, indoor air quality, and infections. Given that this study did not include personal or indoor measurements of MF, we could not identify MF exposures from other sources such as household appliances. Such unmeasured MF exposure may have reduced the statistical power to detect an association in our analysis, but it is unlikely to be associated with exposure from power lines, and thus, we do not expect it to have biased our results. We cannot exclude the possibility that differences in exposure assessment contributed to the differences between our findings and those of Li et al., but we note that in studies of childhood leukemia our exposure assessment method produced results similar to the results of studies using measured fields.

The main limitation of this study is an unexpectedly small number of homes with MF exposure, which allowed us to evaluate cut points used by Li *et al*., but not higher cut points. Also, similar to Li *et al*., we included children who developed asthma in early life in our case definition. Asthma can be difficult to diagnose in very young children because many other respiratory conditions that are common in early life have asthma-like symptoms [24,25]. Therefore, excluding asthma diagnoses prior to age 3 years may have increased the specificity of our outcome definition if our sample had included more exposed cases.

Based on prior studies of residential MF exposure from power lines, we expected to find at least 1% of mothers in our study to have exposure to MF from power lines during pregnancy at a level of at least 0.2 μT [26–29], which would have given us ample statistical power. Surprisingly, the actual percentage of women exposed at this level in our study was only 0.05%. A study by Pedersen *et al*. of childhood leukemia and residential history near 50–400 kV facilities in Denmark reported a 0.5% prevalence of exposure to MF at 0.1–0.4 μT and 0.2% prevalence of exposure above 0.4 μT [28]. We employed the same methodology to estimate calculated fields from power lines. The discrepancy in exposure prevalence between our study and the study by Pedersen *et al*., may be related to the differences in the study time period. While Pedersen *et al*. calculated MF exposure levels for residences dating as far back as 1968, the exposure time period in our study began in 1996. In recent decades, Danish utilities have limited the construction of new high voltage power lines in close proximity to residences, and they have replaced many overhead power lines in residential areas with underground cables. These changes are likely to have had a large impact on reducing residential exposure overall. Finally, our study did not include powerlines carrying 50-132KV, since they were not available for the study due to lack of a centralized data source of information for this power range.

About 60% of pregnant women that were originally invited to participate in the DNBC ultimately enrolled in the study. Authors have previously examined the potential for participation-related biases in the DNBC, with some indication that effects of selection bias are likely to be small [30]. Therefore, we do not expect selection bias to have had a major impact on our results. Loss-to-follow-up in the cohort between the time of enrollment and later stages of data collection may be more likely in women with a low social occupational status [31]. However, our exposure data were obtained from the utility company, and most of our outcome data were obtained from population registers (DNPR and RMPS). As these data were collected without the need for participant contact, loss-to-follow-up is unlikely to have biased our findings.

RMPS data in Denmark are maintained by and accessed through Statistics Denmark, the central authority on statistics about the Danish population. In order to protect individual confidentiality, Statistics Denmark regulations prohibit the reporting of any results from cell sizes less than n = 5. Several of the analyses we originally planned to carry out were not feasible due to the small number of exposed residences in our dataset. In accordance with the regulations, we did not report results from analyses of more specific time windows of exposure (e.g., during pregnancy only) or of more specific asthma case definitions (e.g., cases diagnosed after age 3 years) due to very small analysis cell sizes. However, those results did not meaningfully add to our reported findings or alter our interpretation of them.

We did not find evidence to suggest that residential exposure to MF during pregnancy or early childhood increased the risk of childhood asthma. This interpretation is in line with the lack of an established biological mechanism directly linking MF exposure to asthma.
